# PDB‐tools web: A user‐friendly interface for the manipulation of PDB files

**DOI:** 10.1002/prot.26018

**Published:** 2020-11-07

**Authors:** Brian Jiménez‐García, João M. C. Teixeira, Mikael Trellet, João P. G. L. M. Rodrigues, Alexandre M. J. J. Bonvin

**Affiliations:** ^1^ Bijvoet Centre for Biomolecular Research Utrecht University Utrecht The Netherlands; ^2^ Program in Molecular Medicine The Hospital for Sick Children Toronto Ontario Canada; ^3^ Department of Structural Biology Stanford University School of Medicine Stanford California USA

**Keywords:** bioinformatics, PDB, structural biology, web server

## Abstract

The Protein Data Bank (PDB) file format remains a popular format used and supported by many software to represent coordinates of macromolecular structures. It however suffers from drawbacks such as error‐prone manual editing. Because of that, various software toolkits have been developed to facilitate its editing and manipulation, but, to date, there is no online tool available for this purpose. Here we present PDB‐Tools Web, a flexible online service for manipulating PDB files. It offers a rich and user‐friendly graphical user interface that allows users to mix‐and‐match more than 40 individual tools from the *pdb‐tools* suite. Those can be combined in a few clicks to perform complex pipelines, which can be saved and uploaded. The resulting processed PDB files can be visualized online and downloaded. The web server is freely available at https://wenmr.science.uu.nl/pdbtools.

## 1 INTRODUCTION

The Protein Data Bank (PDB) format, which was created in 1976 to allow researchers to store and share 3D structures, remains a popular file format used by many software to represent coordinates of macromolecular structures such as proteins or nucleic acids,^1^ even though the macromolecular Crystallographic Information Framework (mmCIF) dictionary^2^ is now the standard for the worldwide PDB (wwPDB).^3^ Understanding how the PDB file format remains in use after four decades and several technological leaps requires traveling back to the time of its inception. Despite multiple changes and revisions over the years, the core of the PDB format remains a series of lines limited to 80 characters in length, a leftover requirement from the computer punch cards used to exchange the atomic coordinates in the early days of structural biology. Each line in a PDB file refers to a specific type of record, such as coordinates or details on the experimental setup. Each record then stores data in multiple fixed‐width columns as plain text, lending very easily to visual inspection and editing.

Other file formats were developed in the past decades to overcome the limitations of the PDB format. The Crystallographic Information File (CIF) was proposed in 1991^4^ by a working group from the International Union of Crystallography (IUCr) to support electronic publication of small molecule crystal structures. Later, this format was adapted for larger macromolecules, creating the mmCIF format.^2^ The concept behind mmCIF files is a dictionary of data items describing macromolecular structure and information of macromolecular crystallographic experiments. Unlike the PDB format, mmCIF files store data in variable‐width fields and as such, there are no limits to the number of atoms, residues, or chains.

In July 2019, the mmCIF format was adopted as the official format for structural data in the worldwide PDB database (wwPDB).^5^ Despite this change, the PDB format remains very much in use as the de facto file format for a large variety of structural calculation, modeling, and analysis software.

Naturally, such a long‐lived and important file format gave rise to a wide and colorful variety of parsing and editing software toolkits written in nearly all popular general‐purpose programming languages. Examples of such toolkits include *Bioperl* ^6^, *BioJava* ^7^, *BioPython* ^8^, ^9^, *BioRuby* ^10^, *BioJulia* ^11^, and *ESBTL* ^12^, for the Perl, Java, Python, Ruby, Julia, and C++ programming languages respectively. Other recent contributions include *atomium* ^13^ and *Biotite* ^14^, both written in Python. In addition to these computational frameworks, molecular visualization software such as UCSF Chimera,^15^ ChimeraX,^16^ or PyMOL,^17^ offer powerful parsing and editing capabilities through user‐friendly graphical interfaces that require little to no programming knowledge. Operating on large collections of PDB files, however, particularly in high‐performance computing environment and pipelines, requires solutions in between fully‐fledged software libraries and graphical interfaces. One such solution is the *pdb‐tools* project,^18^ a set of dependency‐free command line tools written in Python and similar in philosophy to the GNU core utilities: Each tool was designed to perform a simple operation on a given PDB input, but multiple tools can be chained together in complex pipelines. The *pdb‐tools* can download both PDB and mmCIF files from the wwPDB database, interconvert between the two formats, and perform a variety of selection, editing, and validation routines. This approach has been shown to be very useful for the structural biology community, judging by the popularity of the package on PyPI (https://pypistats.org/packages/pdb-tools) and the number of clones and forks of the public repository (https://github.com/haddocking/pdb-tools).

Our team develops several widely‐used web servers, among which HADDOCK for integrative modeling of biomolecular complexes,^19^, ^20^ DisVis^21^ for explorative modeling of protein complexes, and PowerFit^22^ for rigid‐body fitting in cryo‐EM density maps.^23^ In our experience as developers, users and educators, researchers tend to favor using a web interface over downloading, compiling, and installing software. Indeed, despite increasing levels of literacy in programming and computing,^24^ many users remain unfamiliar or uncomfortable using command‐line interfaces and/or a GNU/Linux operating system. As such, we developed a web version of *pdb‐tools* (PDB‐Tools Web). Our web server offers a rich and user‐friendly graphical user interface that allows users to mix‐and‐match the different tools and perform complex pipelines in a few clicks. The web server is available at https://wenmr.science.uu.nl/pdbtools.

## 2 IMPLEMENTATION

### 2.1 Code development

The PDB‐Tools Web interface is available to researchers for free and does not require registration. It uses the Python 3 Flask framework (https://flask.palletsprojects.com, version 1.1.1) and the Jinja (https://jinja.palletsprojects.com, version 2.10.3) templating language for server‐side logic, and JavaScript for client‐side logic. The web server is running alongside the other web portals operated under wenmr.science.uu.nl.

In addition to developing the web server, we set up a support forum (https://ask.bioexcel.eu/c/pdb-tools) where users can easily ask questions, report problems, provide suggestions, and give feedback. Having PDB‐Tools Web endorsed by the BioExcel consortium, which also operates forums for HADDOCK, DisVis and PowerFit, ensures a large visibility and a proof of its usefulness for the molecular modeling community.

### 2.2 Molecular Visualization

We used the *NGL Viewer* v2.0.0^25^, ^26^ JavaScript package to implement an on‐the‐fly molecule visualizer that users can use to inspect the PDB before and after executing the pipeline. This visualizer includes several features, such as showing molecular surfaces or water and ion molecules, that users can be enable or disable at will.

### 2.3 Limitations

Because PDB‐Tools Web server depends on the original *pdb‐tools*, it is constrained to the input/output data formats provided by the latter. In addition, since all *pdb‐tools* (except the converters) read and write PDB files, our web service is unable to handle very large structures that are available only in mmCIF format, for example, complete ribosomes. Generally, mmCIF files provided by the user will be first converted to PDB format by the *pdb_fromcif* script. If this conversion is not possible, the server will alert the user to the issue and halt execution of the pipeline. Finally, the user has the option to download the results of his pipeline in either PDB or mmCIF format.

### 2.4 Documentation

The server includes a *manual* section that includes detailed documentation on how to use the server and includes a table describing all the available tools (Table 1).

## 3 RESULTS AND DISCUSSION

By integrating the *pdb‐tools* package with modern web technologies, as described in the section above, our PDB‐Tools Web server provides a straightforward and powerful interface to manipulate both individual PDB files and (compressed) archives. The landing page offers a quick summary of the functionalities of the service and two main entries, one pointing to a pre‐calculated example, and another allowing users to submit a new pipeline. In addition, the navigation bar provides links to the manual and help pages.

**FIGURE 1:**
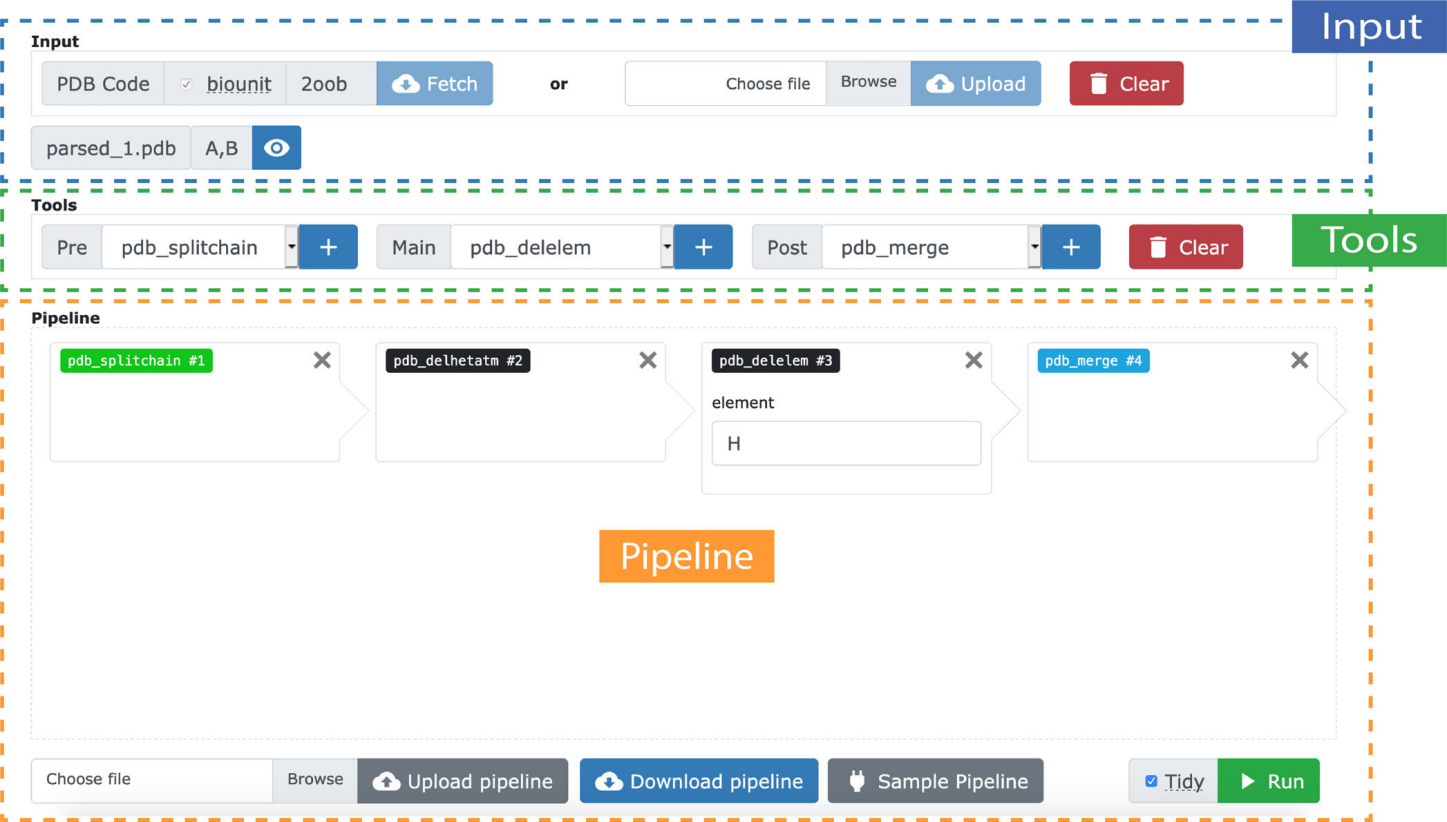
View of the submission interface. The PDB‐Tools Web submission interface is composed of three main sections: (1) Input of the service, (2) available tools, and (3) pipeline canvas and controls

The submit page is a fully‐featured interface providing access to all possible actions when setting up a pipeline and is organized as follows. There are three main sections: *Input*, *tools* and *pipeline* (Figure 1).

**TABLE I:** List of all *pdb‐tools* included in the web server

Tool name	Description
pdb_splitchain*	Splits a PDB file into several, each containing one chain.
pdb_splitmodel*	Splits a PDB file into several, each containing one MODEL.
pdb_splitseg*	Splits a PDB file into several, each containing one segment.
*pdb_b*	Modifies the temperature factor column of a PDB file (default 10.0).
*pdb_chain*	Modifies the chain identifier column of a PDB file (default is an empty chain).
*pdb_chainxseg*	Replaces the segment identifier column by the value in the chain identifier column of the PDB file.
*pdb_chkensemble*	Performs a basic check on a multi‐model PDB file (ensemble) to ensure all models have exactly the same atoms.
*pdb_delchain*	Deletes all atoms matching specific chains in the PDB file.
*pdb_delelem*	Deletes all atoms matching the given element in the PDB file. Elements are read from the element column.
*pdb_delhetatm*	Removes all HETATM records in the PDB file.
*pdb_delinsertion*	Deletes insertion codes in a PDB file, shifting the residue numbering of downstream residues. Allows for picking specific residues to delete insertion codes for.
*pdb_delres*	Deletes a range of residues from a PDB file. The range option has three components: start, end, and step. Start and end are optional and if omitted the range will start at the first residue or end at the last, respectively. The step option can only be used if both start and end are provided. Note that the start and end values of the range are purely numerical, while the range actually refers to every N‐th residue, regardless of their sequence number.
*pdb_delresname*	Removes all residues matching the given name in the PDB file. Residues names are matched without taking into consideration spaces.
*pdb_element*	Assigns the element column to the PDB file, guessing the element from the atom.
*pdb_gap*	Detects gaps between consecutive residues in the sequence, both by a distance criterion or discontinuous residue numbering. Only applies for protein residues.
*pdb_head*	Returns the first N coordinate (ATOM/HETATM) lines of the file.
*pdb_keepcoord*	Removes all non‐coordinate records from the file. Keeps only MODEL, ENDMDL, END, ATOM, HETATM, and CONECT.
*pdb_occ*	Modifies the occupancy column of a PDB file (default 1.0).
*pdb_reatom*	Renumbers atom serial numbers of the PDB file starting from a given value (default 1).
*pdb_reres*	Renumbers the residues of the PDB file starting from a given number (default 1).
*pdb_rplchain*	Performs in‐place replacement of a chain identifier by another.
*pdb_rplresname*	Performs in‐place replacement of a residue name by another. Affects all residues with that name.
*pdb_seg*	Modifies the segment identifier column of a PDB file (default is an empty segment).
*pdb_segxchain*	Replaces the chain identifier column by the value in the segment identifier column of the PDB file. Truncates the segment identifier if longer than one character.
*pdb_selaltloc*	Picks one location for each atom with fractional occupancy values.
*pdb_selatom*	Selects all atoms matching the given name in the PDB file. Atom names are matched without taking into consideration spaces, so ‘CA ‘(alpha carbon) and ‘CA ‘(calcium) will both be kept if ‐CA is passed.
*pdb_selchain*	Extracts one or more chains from a PDB file.
*pdb_selelem*	Selects all atoms that match the given element(s) in the PDB file. Elements are read from the element column.
*pdb_selhetatm*	Selects all HETATM records in the PDB file.
*pdb_selres*	Extracts residues from a PDB file, either arbitrarily or in a range. The range option has three components: start, end, and step. Start and end are optional and if omitted the range will start at the first residue or end at the last, respectively.
*pdb_selresname*	Selects all residues matching the given name in the PDB file. Residues names are matched without taking into consideration spaces.
*pdb_selseg*	Extracts one or more segments from a PDB file based on their segment identifiers.
*pdb_shiftres*	Renumbers the residues of the PDB file by adding/subtracting a given number from the original numbering.
*pdb_sort*	Sorts the ATOM/HETATM/ANISOU/CONECT records in a PDB file.
*pdb_tidy*	Modifies the file to adhere (as much as possible) to the format specifications.
*pdb_tocif*	Converts a PDB file to the mmCIF format.
*pdb_tofasta*	Extracts the residue sequence in a PDB file to FASTA format.
*pdb_validate*	Validates the PDB file ATOM/HETATM lines according to the format specifications.
*pdb_wc*	Summarizes the contents of a PDB file, like the wc command in UNIX.
*pdb_merge* ^*#*^	Merges several PDB files into one. The contents are not sorted, and no lines are deleted (eg, END, TER statements) so we recommend combining it with pdb_tidy.
*pdb_mkensemble* ^*#*^	Merges several PDB files into one multi‐model (ensemble) file. Strips all HEADER information and adds REMARK statements with the provenance of each conformer.

*Note:* For each of the tools a short description is provided. Tools flagged with a “*” are pre‐processing tools, usable only at the beginning of the pipeline. Those with a “*#*” are post‐processing tools; only one of those can be used at a time and always with a previous pre‐processing tool selected, for example, for merging previously split structures.

The *input* section prompts users to either provide a PDB ID code, which triggers a download from the database, or upload a file from their computer. After the structure is loaded, a small panel pops up, providing general information about the input molecule to which users can refer during the pipeline setup. Clicking the eye‐shaped button opens the molecular visualization panel. Finally, a *clear* button resets the page to its initial state, discarding all previously uploaded input data and associated results.

The *tools* section is composed of three dropdown menus corresponding to pre‐processing, manipulation and post‐processing actions respectively. In short, the user can browse the available actions through the different dropdown menus and add them to the pipeline using the “*+*” button. Some actions accept options, for example, a chainID for *pdb_selchain*, which the user can input directly in the pipeline canvas. Users can clear the current pipeline via the “*clear* “button on the right of this section, without losing the information provided as *input*.

The pipeline section is further sub‐divided in a canvas that displays the current state of the pipeline, and a bottom section with buttons to load and save pipelines. Users can also save their pipeline (in JSON format) using the button “*Download pipeline*” at the bottom of the pipeline canvas. Together with the possibility to load a pipeline, through the “*Upload pipeline*” button, this feature allows users to reproduce previous pipelines with minimal effort. Finally, users may load an example input and pipeline using the button “*Sample pipeline*”.

A few implementation details are worthy of note. First, while the first selected action will always act on the input structure, consecutive actions will act on the result of the previous action. In addition, by definition, some tools can only be used at the beginning or end of a pipeline. For example, *pdb_splitchain* splits the input structure into several files, each containing an individual chain. After this action, all subsequent steps in the pipeline apply individually to all chains. On the other hand, users can use *pdb_merge* to combine all the separate files again into one single structure. Alternatively, users can simply download each individual chain.

The bottom of the submit page shows a “*Tidy*” option that, if checked, will run the *pdb_tidy* tool to process the output to adhere (as much as possible) to the PDB format specifications. A green “*Run*” button submits the pipeline to the server and executes it on the input file. The user is then redirected to a results page, where they can view and download the resulting structure and accessory output files. As expected, the output generated by *pdb‐tools* and the web server can be used as input itself on another run. If a similar or identical pipeline using the same input of a previous job is required, there is a button in the results page “*Resubmit this pipeline*” which will facilitate this action. This might be useful for example to apply a similar pipeline to another chain of the same input PDB file.

The example page presents the result of a pre‐calculated pipeline submission, allowing users to experience the look and feel of a real submission.

Finally, since the first version of our web server was made available online, in March 2020, it has processed more than 1200 individual jobs and received very good feedback from our users with an average score of 4.95 on a scale from 1 (worst) to 5 (best) based on 58 respondents.

## 4 CONCLUSIONS

In this manuscript, we present PDB‐Tools Web, an online tool to manipulate PDB files with a modern interface and a rich user experience. Our server is freely available and does not require registration from users. Our target audience are users that cannot, or prefer not to, install software locally. In addition, we have documented all of the server’s main features exhaustively in the user manual and we offer user support through the ask.bioexcel.eu platform. We also linked the web server to our education and tutorials portal (http://www.bonvinlab.org/education/), as the tool of choice for manipulating PDB files. Finally, we constantly collect feedback to gauge user satisfaction and help improve our service.

In summary, we believe that PDB‐Tools Web should be a valuable resource for many students and researchers in computational structural biology, further contributing to closing the existing gap between powerful command‐line toolkits and user‐friendly interfaces.

5

### PEER REVIEW

The peer review history for this article is available at https://publons.com/publon/10.1002/prot.26018.
